# Parallel analysis of global garlic gene expression and alliin content following leaf wounding

**DOI:** 10.1186/s12870-021-02948-0

**Published:** 2021-04-10

**Authors:** Xuqin Yang, Yiren Su, Jiaying Wu, Wen Wan, Huijian Chen, Xiaoying Cao, Junjuan Wang, Zhong Zhang, Youzhi Wang, Deliang Ma, G. J. Loake, Jihong Jiang

**Affiliations:** 1 The Key Laboratory of Biotechnology for Medicinal Plant of Jiangsu Province, School of Life Science, Jiangsu Normal University, Xuzhou, 221116 Jiangsu China; 2 XuZhou Nuote Chemical co., Ltd., Xuzhou, 221137 Jiangsu China; 3 Institute of Molecular Plant Sciences, School of Biological Sciences, University of Edinburgh, Edinburgh, EH9 3JH UK

**Keywords:** Garlic, *Allium sativum*, Transcriptomics, Alliin, Gene prediction

## Abstract

**Background:**

*Allium sativum* (garlic) is an economically important food source and medicinal plant rich in sulfides and other protective substances such as alliin, the precursor of allicin biosynthesis. Cysteine, serine and sulfur is the precursor of alliin biosynthesis. However, little is known about the alliin content under abiotic stress or the mechanism by which it is synthesized.

**Results:**

The findings revealed that the content of alliin was lowest in the garlic roots, and highest in the buds. Furthermore, alliin levels decreased in mature leaves following wounding. Transcriptome data generated over time after wounding further revealed significant up-regulation of genes integral to the biosynthetic pathways of cysteine and serine in mature garlic leaves.

**Conclusions:**

The findings suggest that differential expression of cysteine, serine and sulfide-related genes underlies the accumulation of alliin and its precursors in garlic, providing a basis for further analyses of alliin biosynthesis.

**Supplementary Information:**

The online version contains supplementary material available at 10.1186/s12870-021-02948-0.

## Background

Garlic *(Allium sativum L.*), a diploid (2n = 2x = 16) plant species and one of the most medicinally and economically important members of the Allium genus, has been widely cultivated for more than 5000 years [1–3]. Studies have shown a number of biological functions from strong antioxidant potential [4], stabilization of blood pressure [5], reduced cancer risk [6], cardiovascular protection [7], and reduced hyperlipidemia [8], with the roots, bulbs, leaves and sprouts all possessing important agronomical traits. Of these, the bulbs, which consist of several abnormal axillary buds in a characteristic clove shape, are the most economically important [2]. A key feature of garlic is the production of secondary metabolites rich in sulfur, such as S-methyl-L-cysteine sulfoxide (MCSO, methiin), S-propyl-L-cysteine sulfoxide (PCSO, propiin), S-trans-1-propenyl-L-cysteine sulfoxide (PECSO, isoalliin) and, most importantly, S-allyl-L-cysteine sulfoxide (ACSO, alliin) [9]. These sulphur compounds are also taste precursors, regulating the flavour and odour of the developing bulbs [10]. Allicin, which is derived from alliin, is another sulfur compound and the key bioactive molecule in garlic [11, 12]. Various in vivo and in vitro studies have demonstrated the anti-apoptosis and anti-oxidation potential of allicin [13]. However, allicin is also extremely unstable at room temperature due to its rapid decomposition into diallyl disulfides and sulfur dioxide [14]. Allicin is produced from alliin, which is located in the cytoplasm, via alliinase [15], which is located in the vacuole. Accordingly, when cells are broken or damaged, allinase is released, inducing the conversion of alliin into allicin [16, 17].

Alliin is the most abundant non-protein sulfur-containing amino acid in fresh garlic, accounting for more than 90% of its sulfur compounds [18]. Stable and odorless molecule [19], alliin is of significant nutritional and medicinal value [20], exhibiting antioxidant [21] and anti-inflammatory [22] activity, promoting cardiovascular function [23] and conveying beneficial effects on certain intestinal diseases [24]. Alliin is synthesized in the leaves then transferred to the bulbs [25, 26], during which process, the roots absorb sulfates from the soil and transport them to the leaves. These sulfates are then used to synthesis glutathione in the chloroplasts, which is then transferred to the developing bulb via the vascular system. Alliin stored in the leaves is also transported to the sprouts, protecting them from attack by microorganisms and herbivores [27].

Despite the importance of alliin in the commercial potential of garlic, the details of alliin biosynthesis remain largely unknown. Based on the results of radiotracer experiments and chemical analyses [28], it is thought that the alliin biosynthetic pathway involves cysteine. Briefly, the pathway involves S-alk(en)ylation of the cysteine residue in glutathione, followed by the removal of the glycyl group to form an intermediate γ-glutamyl-S-alk(en)yl-L-cysteine. By ASFMO1(AsFMO1 is a flavin-containing monooxygenase, in garlic, which is responsible for the S-oxygenation reaction in the biosynthesis of alliin) catalytic get alliin. However, others also support the involvement of the serine-thiol pathway: bonding of serine with Acetyl CoA to produce *O*- Acetylserine followed by the conjugation of *O*-Acetylserine with the allyl thiol compound *S*-allylcysteine, and lastly, *S*-allylcysteine oxidation of ACSO. While γ-serine acetyl transpeptidase is thought to catalyse the first step, S-cysteine acetyl transpeptidase catalyses step 2, resulting in the formation S-allyl cysteine [29]. Understanding the biosynthetic pathways of both cysteine and serine is therefore important in determining the pathway of alliin biosynthesis.

Garlic (2n = 2x = 16) has a large genome [30]. It is estimated to be 15.9G and sterile cultivars, and therefore, no classical breeding or genetic studies have been carried out [31]. As a result, and despite its agronomic importance, garlic remains largely undomesticated, hampering its commercial potential. Liu [32] recently reported the genome assembly of garlic at the chromosome level, making it the first species within the *Allium* genus to be sequenced. According to these sequencing results, garlic experienced a recent occurrence of burst of transposable elements. Alliinase genes and content are thought to have rapidly expanded during the burst of transposable elements. Which helps explain the evolution of allicin biosynthesis-related genes. Four GSH1-orthologous genes, one GSH2-orthologous gene and one PCS-orthologous gene were also identified, expression of which was up-regulated during garlic bulb development. These genes are therefore potential candidates of the alliin biosynthesis pathway.

Although genome sequencing is an effective tool, it is both time-consuming and costly. In contrast, transcriptome analysis has the advantages of high speed, low cost and the absence of limitations in terms of genome complexity [33]. In recent years, transcriptomics has been used in correlation analysis of different traits, aiding studies of genetic association, notably in complex polyploid species [34–36], such as *Salvia miltiorrhiza* [37], *Pinellia ternata* [38], and *Cicer arietinum L.* [39] Meanwhile, Jun [2] carried out transcriptome analyses of garlic bulbs, and identified 22 candidate transcripts with complex interactions. Mitrová [40] through transcriptome analysis also revealed the corresponding genetic changes in allinase during the whole cycle, while reported that the transcription of two particular enzymes was highest during sprouting. Moreover, Einat [30] combined transcriptome and proteome analyses of garlic flowers and pollen revealed potential molecular markers for male fertility and sterility. Liu [41] also carried out association mapping of three yield traits in the garlic bulb: bulb weight, bulb diameterand garlic quantity, revealing 17 significantly correlated single nucleotide polymorphisms (SNPs).

However, despite the above studies, transcriptome analysis of alliin biosynthesis in garlic leaves after wounding have yet to be carried out. In this study, we carried out transcriptome analysis of the key pathways thought to be involved in alliin metabolism over time after wounding. The findings provide a basis for the mechanism of alliin biosynthesis under physical stress.

## Results

### Measurement of alliin content

To determine the differences in alliin content among the garlic cloves, inner buds, roots, and sprouts, a post-column derivatization method using ninhydrin was established. The findings revealed that the bulbs contain the highest level of alliin (1.474 mg.ml^-1,^ DW), while the roots contain the lowest (0.019 mg.ml^-1,^ DW).

**Fig. 1:**
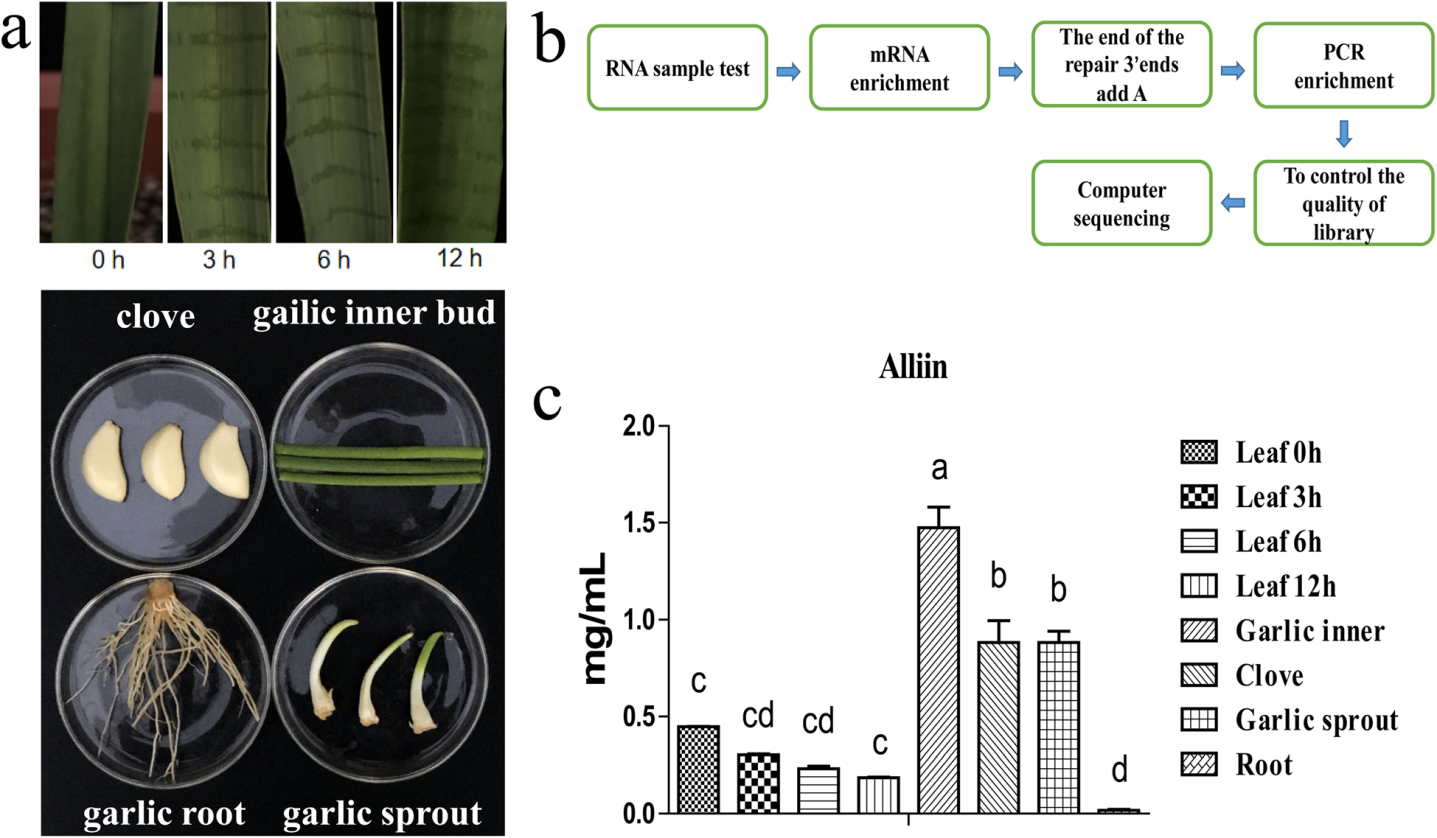
Variation in alliin contents among samples. **a** Mature garlic tissue: root, bulb, leaf, inner bud, and sprout. Fresh tissue samples were collected. **b** The process of transcriptome sequencing. **c** S-4330D was used to measure the alliin contents. Different letters indicate a significant difference between samples (*P* < 0.05)

Next, to determine the alliin content of the garlic leaves after wounding, damaged leaves were sampled at different time points after injury then analysed using the same method. Three biological replicates were sampled at each time point (T1/3, T4/6, T7/9, T10/12 represents 0, 3, 6, 12 h’s wounding treatment, respectively). As shown in Fig. 1c, the content of alliin decreased over time in the wounded leaves.

### Transcriptome analysis of the garlic leaves after wounding

**Fig. 2:**
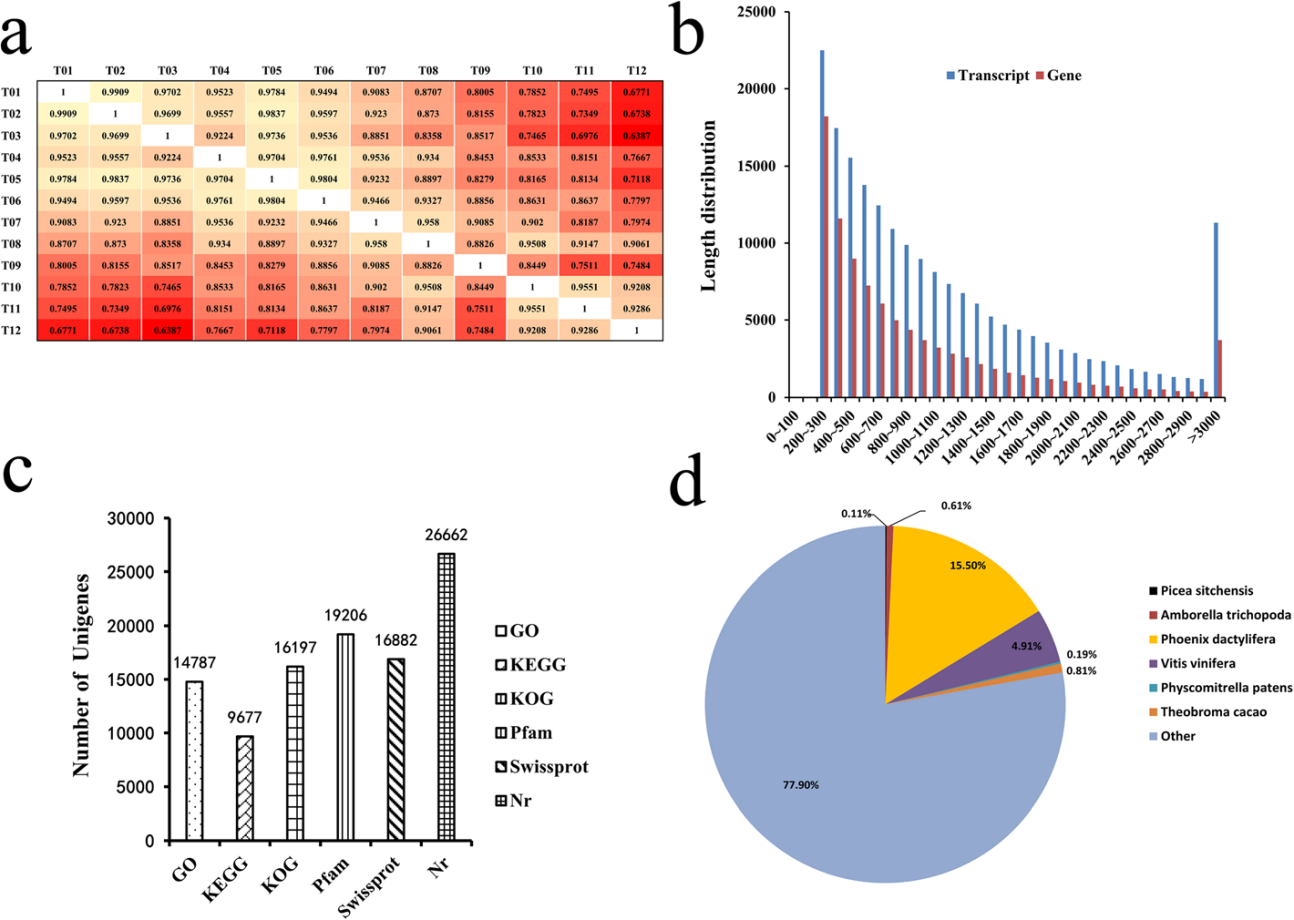
Results of transcriptome analysis. **a** Biological repeat correlations between the garlic sequencing data were determined with Pearson’s correlation coefficients. **b** Scatter diagram showing the degree of gene correlations between samples. **c** Size distributions of the transcripts and unigenes. **d** Unigene annotation based on various databases. e) Unigene species distribution

Approximately, 0.03% of the raw reads, including low quality and short reads, were removed post filtering of the adapter sequences, resulting in 34,855,947 clean reads for T1/3, 30,830,543 for T4/6, 29,001,419 for T7/9 and 34,278,922.33 for T10/12 (Additional file 1). Pair-wise Pearson’s correlation coefficients of the three replicates × four garlic samples indicated high repeatability of the sequencing data (Fig. 2a). At the same time, comparisons of individual genes between the control group and each treatment group revealed significant differences in reliability among genes.

All reads were assembled using Trinity [42], resulting in 194,627 transcripts (N50:1667) with a mean length of 1157.22 bp (Fig. 2b), and 94,144 unigenes (N50:1394) with a mean length of 933.08 bp. The size distributions of the unigenes and transcripts are shown in Fig. 2b. Overall, 15.38% of the reads were > 2000 bp in length, while 28.78% were < 1000 bp in length. A minimum read length of 200–300 bp occurred in 11.56% of the transcripts, and only 10.36% of the unigenes were > 2000 bp in length, the majority being between 200 and 500 bp. A total of 16,882 unigenes were annotated using the Swiss-Prot database, 26,662 unigenes were identified using the Nr database, 19,206 displayed significant similarities with known proteins in the Pfam database, and 9677 and 16,197 were annotated using the KEGG and KOG databases, respectively (Fig. 2c). The distribution of unigene species annotation is shown in Fig. 2d.

### Identification of differentially expressed unigenes (DEGs) in the garlic leaf samples

**Fig. 3:**
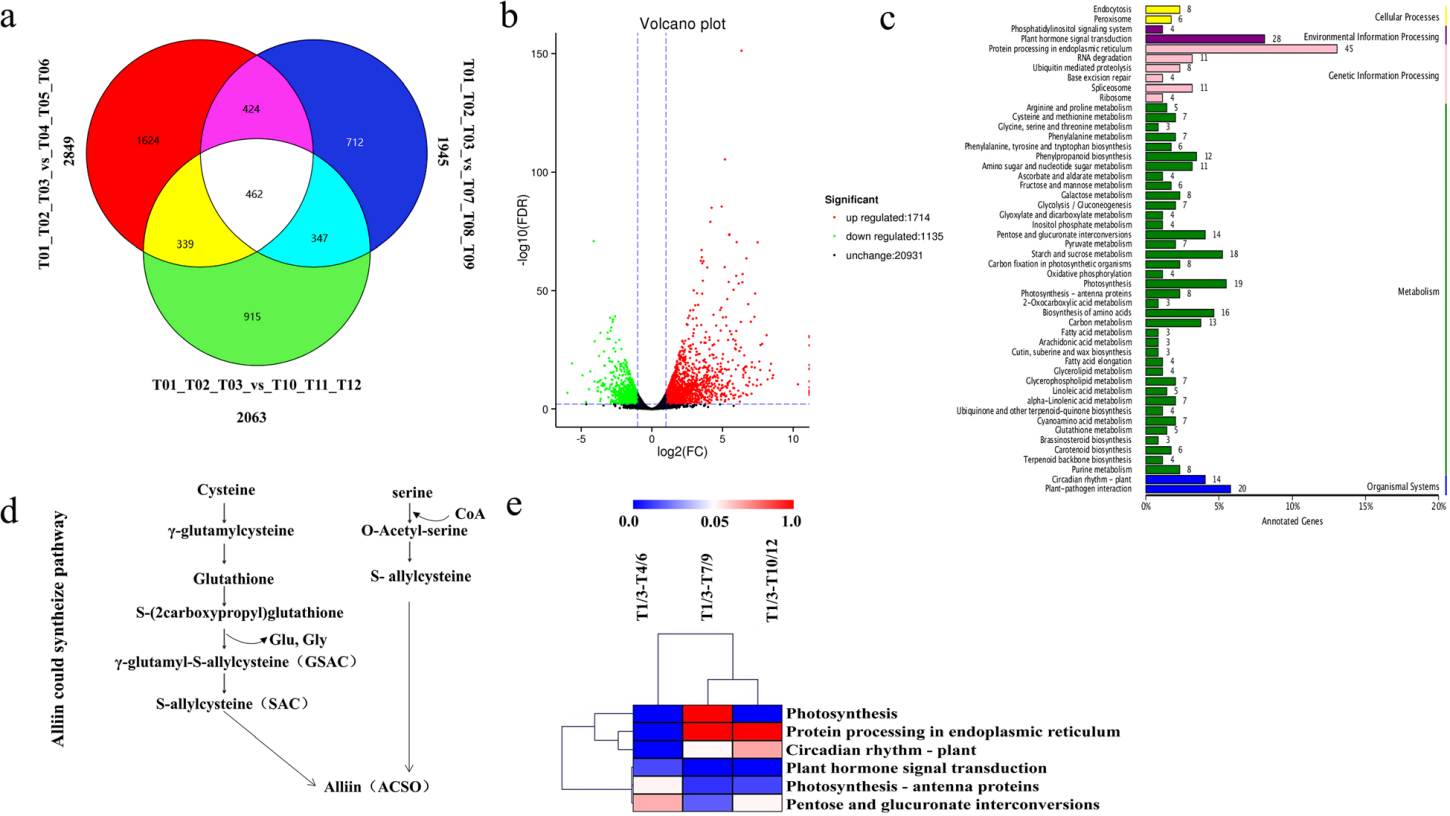
Differentially expressed genes (DEGs) among samples. **a** Venn diagram showing the number of DEGs in each comparison. **b** Volcano plot showing the differences in expression levels and numbers of single genes in each set of comparisons. **c** Annotation results of differentially expressed KEGG genes classified according to the KEGG pathway types of organismal systems, metabolism, cellular processes, environmental information processing, and genetic information processing. **d** Alliin could biosynthesize pathways. **e** KEGG enrichment analysis of each comparison. The heat maps show that all significant *P*-values of KEGG in the three groups of comparisons were represented

A number of the unigenes were classified into KEGG metabolic and signalling pathways. Six KEGG pathways, ‘Protein processing in endoplasmic reticulum’, ‘Plant hormone signal transduction’, ‘Circadian rhythm-plant’, ‘Pentose and glucuronate interconversions’, ‘Photosynthesis - antenna proteins’ and ‘Photosynthesis’, were the most enriched (Fig. 3c; Additional file 2). Venn diagrams were also used to represent the number of differentially expressed genes under each treatment compared with the control (Fig. 3a). By this standard of (*P* < 0.05) in the pathways. Accordingly, 1714 unigenes were found to be up-regulated and 1135 were down-regulated in T1/3 vs. T4/6, while in T1/3 vs.T10/12688 highly-expressed unigenes were up-regulated and 1375 were down-regulated (Fig. 3b; Additional file 3).

Of the these pathways(Fig. 3e), four pathways were significantly enriched (P < 0.05) in T1/3 compared to T4/6. Meanwhile, In the T1/3 compared to T7/9 and T1/3 compared to T10/12, only three pathways were significantly enriched. To predict possible functions and orthology classifications, the unigenes were also compared using the COG database. As a result, 12,375 sequences in T1/3 compared with T4/6 were assigned to 25 COG categories. There is usually only one category of functional prediction. Here, the general functional prediction (2191; 17.71%) represented the largest group followed by replication, recombination and repair (1350; 10.91%); transcription (1099; 8.88%), and signal transduction mechanisms (909; 7.35%). Additional samples used for COG classification are detailed in Additional file 4.

### DEGs associated with the alliin biosynthesis pathway

**Fig. 4:**
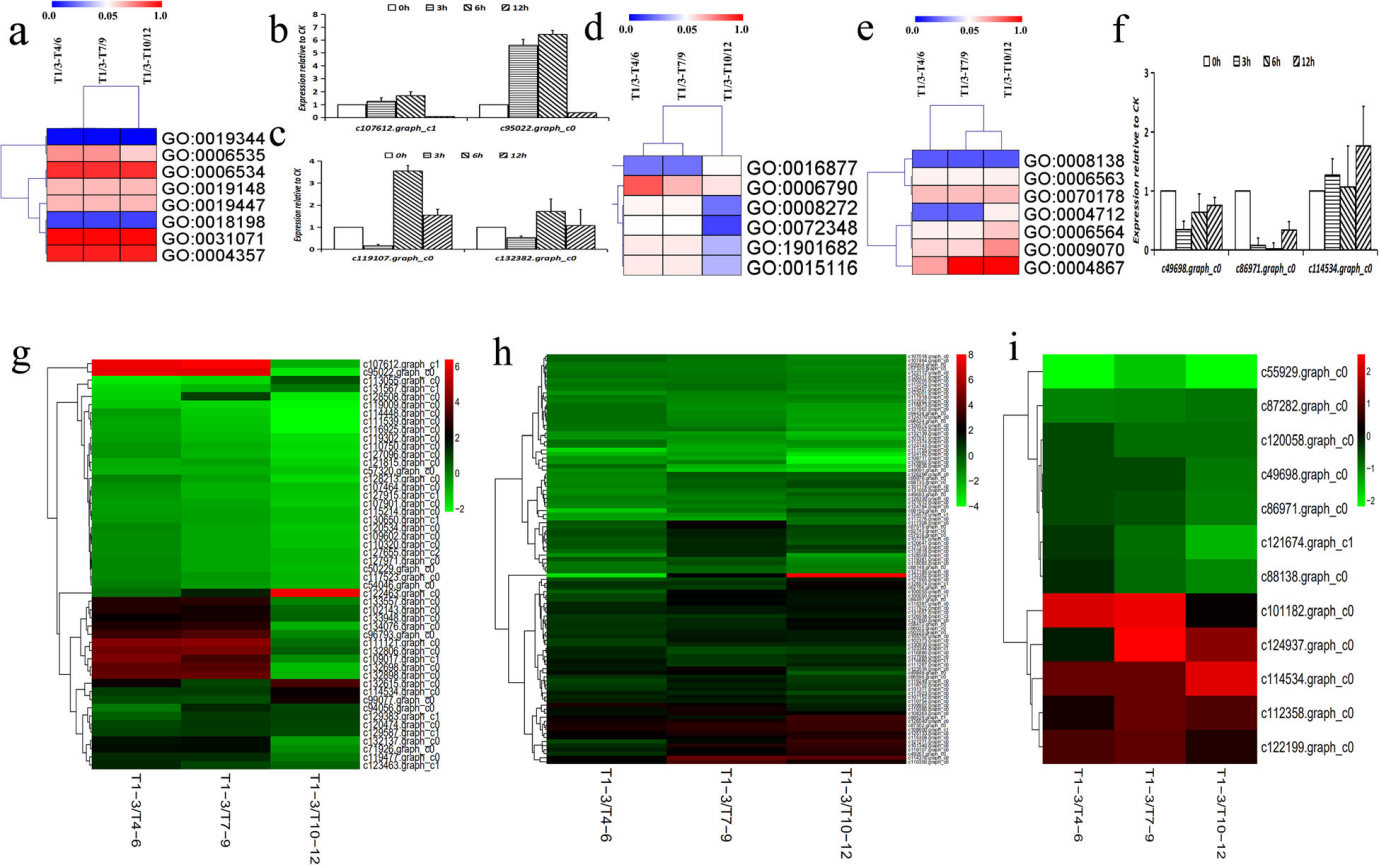
Transcriptome and qRT-pcr analysis of the differences in cysteine, serine and sulfur-related genes. **a** Heat map showing the differences in eight cysteine-related GO terms between T1/3 vs. T4/6, T1/3 vs. T7/9, and T1/3 vs. T10/12. **b** qRT-PCR analysis gene of c107612.graph_c1 and c95022.graph_c0 expressions in 0 h, 3 h, 6 h, and 12 h garlic leaf. 0 h leaf were set as 1.0 in qPCR analysis. **c** qRT-PCR analysis gene of c119107.graph_c0 and c132382.graph_c0 expressions in 0 h, 3 h, 6 h, and 12 h garlic leaf. 0 h leaf were set as 1.0 in qPCR analysis. **d** Heat map showing the differences in six sulfur-related GO terms between T1/3vs. T4/6, T1/3 vs. T7/9 and T1/3 vs T10/12. **e** Heat map showing the differences in seven serine-related Go terms between T1/3 vs. T4/6, T1/3 vs. T7/9, and T1/3 vs. -T10/12. **f** qRT-PCR analysis gene of c49698.graph_c0, c86971.graph_c0, and c114534.graph_c0 expressions in 0 h, 3 h, 6 h, and 12 h garlic leaf. 0 h leaf were set as 1.0 in qPCR analysis. **f** Heat map showing the expression of genes related to the cysteine signalling pathway. **g** Heat map showing the expression of genes related to the serine signalling pathway. **h** Heat map showing the expression of genes related to the sulfur signalling pathway

Detailed steps of the alliin biosynthesis pathway are currently unknown [43]. However, some of the corresponding molecules have been determined, including its precursors: glutathione, glycine, serine [44], cysteine [45] and sulfur [46], which feed a series of hydrocarbylation, alkylation and oxidation reactions (Fig. 3d). In this study, transcriptome analysis revealed five alliin biosynthesis-related GO terms: ‘sulfur compound biosynthetic process’ (GO:0044272), ‘sulfur amino acid metabolic process’ (GO:0000096), ‘cysteine biosynthetic process’ (GO:0019344), ‘L-serine biosynthetic process’ (GO:0006564) and ‘glutathione peroxidase activity’(GO:0004602), providing a basis for further analysis of the differential expression of alliin biosynthesis-related genes during leaf wounding. After wounding, genes in the GO:0044272 pathway, such as c119107.graph_c0 and c132382.graph_c0, significantly changed gradually over time. Moreover, genes encoding sulfur compounds (c49698.graph_c0, c86971.graph_c0, and c114534.graph_c0) were expressed at high levels. The most highly expressed were involved in cysteine biosynthesis: c107612.graph_c1 and c95022.graph_c0. To prove the significantly changed of these seven genes. We conducted qPCR experiments, and the results showed a positive correlation with the heat map.(Fig. 4 b,c,f).

### Transcriptome analysis of the differences in cysteine pathway-related genes

Based on transcriptome analysis, eight cysteine-related GO terms, ‘D-cysteine catabolic process’ (GO:0019447), ‘cysteine biosynthetic process’ (GO:0019344), ‘cysteine biosynthetic process from serine’ (GO:0006535), ‘cysteine desulfurase activity’ (GO:0031071), ‘D-cysteine desulfhydrase activity’ (GO:0019148), ‘glutamate-cysteine ligase activity’ (GO:0004357), ‘peptidyl-cysteine modification’ GO:0018198, and ‘cysteine metabolic process’ (GO:0006534) were identified. Significant differences in GO:0019344 were observed in the T1/3 vs. T4/6, T1/3 vs. T7/9 and T1/3 vs. T10/12 comparisons at 0.00168, 0.0013 and 0.00012, respectively. Meanwhile, significant differences in with GO:0006535 were also observed between the T1/3 vs. T4/6, T1/3 vs. T7/9 and T1/3 vs. T10/12 comparisons at 0.46558, 0.45829 and 0.23682, respectively. Differences between T1/3, T4/6, T1/3, T7/9, T1/3 and T10/12 in terms of cysteine related genes are shown in Fig. 4a and Additional file 5.

The alliin synthesis pathway is thought to involve the cysteine pathway, hence the above difference in related differential genes. With time after wounding, the enrichment of different genes in the cysteine synthesis pathway became increasingly significant, further confirming the involvement of cysteine in alliin synthesis. We also created a heat map based on the differential genes related to cysteine biosynthesis, further highlighting the differences between samples (Fig. 4g, Additional file 6).

### Transcriptome analysis of the differences in serine pathway-related genes

Serine is also thought to play an important role in the alliin synthesis pathway [29]. Based on transcriptome analysis, seven serine-related GO terms were identified: ‘protein serine/threonine/tyrosine kinase activity’ (GO:0004712), ‘protein tyrosine/serine/threonine phosphatase activity’ (GO:0008138), ‘L-serine biosynthetic process’ (GO:0006564), ‘L-serine metabolic process’ (GO:0006563), ‘serine-type endopeptidase inhibitor activity’ (GO:0004867), ‘serine family amino acid biosynthetic process’ (GO:0009070), and ‘D-serine metabolic process’ (GO:0070178). Significant differences in GO:0008138 were observed in all comparisons (Fig. 4e), while significant differences in GO:0004712 were observed between T1/3 vs. T4/6 and T1/3 vs. T7/9. Significant differences in GO:0008138 were also observed between T1/3 vs. T4/6, T1/3 vs. T7/9, and T1/3 vs. T10/12 at 0.01758 and 0.01673 and 0.01689, respectively. In contrast, significant differences in GO:0004712 were observed only between T1/3 vs. T4/6 and T1/3 vs. T7/9 at 0.01939 and 0.01911, respectively. Differences between T1/3, T4/6, T1/3, T7/9, T1/3, and T10/12 in terms of serine-related genes are shown in Additional file 7. As above, heat maps of the serine metabolism-related genes were also created (Fig. 4h; Additional file 8).

### Transcriptome analysis of the differences in sulfur-related diffeences genes

In garlic, sulfur compounds are the most important organic compound. As described earlier, they include alliin, and many other sulfur compounds involved in alliin biosynthesis. Based on transcriptome analysis, six sulfur-related GO terms were identified: ‘sulfate transmembrane transporter activity’ (GO:0015116), ‘sulfate transport’ (GO:0008272), ‘sulfur compound transport’ (GO:0072348), ‘ligase activity, forming carbon-sulfur bonds’ (GO:0016877), ‘sulfur compound transmembrane transporter activity’ (GO:1901682), and ‘sulfur compound metabolic process’ (GO:0006790). Significant differences in GO:0015116 were observed between T1/3 vs. T4/6, T1/3 vs. T7/9, and T1/3 vs. T10/12 at 0.13427 and 0.13008 and 0.03515, respectively (Fig. 4d). In contrast, significant differences in GO:0016877 were only observed between T1/3 vs. T4/6 and T1/3 vs. T7/9 at 0.02281 and 0.02219, respectively. Differences between T1/3, T4/6, T1/3, T7/9, T1/3 and T10/12 in terms of sulfur-related genes are shown in Additional file 9. Heat maps of the sulfur-related genes were also constructed as shown in Fig. 4i and Additional file 10.

### Identification of transcription factor (TF) families in alliin

A number of TFs are thought to play important roles in alliin biosynthesis. In this study, 452 putative TF-encoding genes belonging to 47 major TF families were analysed. Of these, 40 were included in the BHLH family, 36 in the FAR1 family, and 36 in the NAC family. In order to screen key regulators of alliin biosynthesis, eight of these TF were selected for further analysis (Additional file 11).

## Discussion

Allicin, the key sulphoxide in garlic, is produced from the precursor alliin via the action of alliinase. Different tissues and wounded leaves contain various kinds of amino acids, including cysteine, serine and alliin. However, as shown in Fig. 1c, the content of alliin significantly differed between tissues, with the lowest value in the roots and highest in the inner bundle and leaves, consistent with previous findings [25]. At the same time, the content of alliin in the leaves decreased over time after wounding (Fig. 1c). Alliin is synthesised in the cytoplasm, while alliinase is stored in the vacuole. When garlic leaves are damaged, and the cells broken, the alliinase converts alliin into allicin. Thus, the content of alliin in the damaged cell will decrease. Understanding the expression of genes related to the biosynthesis of allicin precursors after wounding stress is therefore helpful in determining the mechanism of alliin biosynthesis.

In this study, a large number of DEGs was identified in comparisons of different time points after wounding (T1/3 vs. T4/6, T1/3 vs. T7/9, and T1/3 vs. T10/12), with enrichment of 12 major pathways. c107612.graph_c1, c95022.graph_c0, c114534.graph_co, c112358.graph_co, and c122199.graph_co were all up-regulated, while c119009.graph_co and c129962.graph_co were down-regulated after wounding as shown in Fig. 3b. Moreover, protein processing differed significantly between T1/3 and T4/6, but not between T1/3 vs. T7/9 and T1/3 vs. T10/12. Cysteine is an effective precursor of alliin biosynthesis, and in this study, according to comparisons of T1/3 vs. T4/6, T1/3 vs. T7/9 and T1/3 vs. T10/12 (Fig. 4b), a large number of DEGs under ‘cysteine biosynthetic process’ were enriched. These findings suggest that cysteine synthesis was activated after wounding. Meanwhile, ‘peptidyl-cysteine modification’ also showed variation, suggesting that a number of peptide groups are also involved in alliin biosynthesis. Based on the above findings, we also created a heat map to further highlight the corresponding differential genes, providing a basis for the role of cysteine-related genes in alliin biosynthesis.

Both sulfur [47] and serine [44] are also precursors of alliin biosynthesis in garlic. Sulfur plays an important role in the synthesis of CSOs, and is an important determinant of flavour in allium plants. Moreover, alliin itself is a sulfide [48]. In this study, expressions of genes in the ‘sulfur compound transmembrane transporter activity’ and ‘sulfate transmembrane transporter activity’ pathways were significantly different between the post-wounding (T4/6, T7/9 and T10/12) and unwounded samples (T1/3). These findings suggest that when the cytomembrane is broken, transport activity of sulfur compounds (including alliin and its precursors) is activated. Expressionsof genes in the ‘protein serine/threonine/tyrosine kinase activity’ and ‘protein tyrosine/serine/threonine phosphatase activity’ pathways were also significantly different between the post-wounding (T4/6, T7/9 and T10/12) and unwounded samples (T1/3). Kinase and phosphatase catalyse two correlated processes. Phosphatase is activated during the cell response to stress, resulting in numerous free hydroxyls and ions, which are then used by kinase to promote the synthesis of alliin and its precursors.

## Conclusions

Details of the alliin biosynthesis pathway remain largely unknown; however, the precursors of alliin in garlic (cysteine, serine and sulfide) have been identified. The metabolic pathways of these precursors are also known, providing a basis for studies of alliin biosynthesis. This study revealed the differential expression of alliin biosynthesis-related genes involved in cysteine synthesis and metabolism, serine synthesis and enzyme activity, and sulfur formation and transport, in garlic leaves after wounding. These findings provide a deeper understanding of the regulation of genes related to alliin biosynthesis, and highlight candidate genes for further analyses in the future.

## Methods

### Plant materials and RNA extraction

Garlic samples were collected form Pizhou, China (abbreviation: PW), and cultivated in the test farm (Xuzhou city, Jiangsu province) in an area of more than 100m^2^. Vernier calipers were used to measure garlic leaf length, width and thickness at maturity in plants with a similar phenotype. Samples were obtained at 0, 3, 6 and 12 h after wounding. Garlic root, clove, inner bud and sprout samples were also collected (Fig. 1a) then immediately frozen in liquid nitrogen and stored at 80 °C until use. Total RNA was extracted using a Plant Total RNA Isolation Kit(Vazyme, China) according to the manufacturer. DNase I (Vazyme, China) was added to the mixture to prevent DNA contamination. Purified RNA was analysed by 1% agarose gel electrophoresis and the quality of total RNAs was confirmed using an RNA 6000 Nano LabChip kit (Agilent, Santa Clara, CA, USA) with an RNA integrity number > 7.0.

### Library preparation and transcriptome analysis

The samples of appropriate quality total RNA of 3 μg from each RNA is prepared(at 0 h, 3 h, 6 h, 12 h after wounding, 3 biological replicates). Library construction was carried out as follows: Oligo (dT) magnetic beads were enriched with eukaryotic mRNA. The mRNA was then randomly fragmented using fragmentation buffer(Biomarker, China) and used as a template for first-strand cDNA synthesis. Buffer, dNTPs, RNase H and DNA polymerase I were then added prior to second-strand cDNA synthesis. AMPure XP beads (Biomarker, China)were used to purify the cDNA, while purified double-stranded cDNA was used to repair and A-tails were added to connect the sequencing beads. AMPure XP beads (Biomarker, China)were then used to select the fragment size before constructing the cDNA library following PCR enrichment. Qubit 2.0(Biomarker, China) and Agilent 2100 (Agilent, USA)were used to determine the library concentration and insert size. To ensure library quality, Q-PCR was used to accurately quantify the effective concentration. HiSeq2500(Illumina, San Diego, USA) was then used for High-throughput sequencing with a reading length of PE125. The raw data was then filtered to obtain high-quality clean reads. Trinity software [42] was used to assemble the clean data for genetic identification and expression analysis at the different treatment group level [43] (Fig. 1b). The raw sequence data has been submitted to the ENA Short Read Archive with accession number PRJEB33852.

### Functional annotation and analysis

An NCBI BLAST search was used to compare unigene sequences with the Non-redundant (NR) Protein, Swiss-Prot, Gene Ontology (GO), Clusters of Orthologous Groups of proteins (COG), EuKaryotic Orthologous Groups (KOG) and Kyoto Encyclopedia of Genes and Genomes (KEGG) databases. KOBAS 2.0 was used for KEGG Orthology annotation, while HMMER and PFAM were used to compare sequences using the annotation information.

### Differentially expressed unigene (DEGs) analysis

Bowtie [11] compared the sequencing reads of each sample with the Unigene library then estimated expression levels using RSEM [12] based on the comparison results. The abundance of corresponding unigenes is indicated by FPKM values. Here, the DEGs were screened according to the following criterion: FDR < 0.01 and a fold-change (FC) ≥ 2. The abundance values of all transcripts were normalized then MultiExperiment Viewer (version 4.9.0) was used to construct heat maps based on the transformation values. In these maps, columns represent different samples and rows represent different genes.

### Homology analysis and CDS predictions

TransDecoder software was used to compare the length of open-reading frames, logarithmic likelihood function values and amino acid sequences with a protein structure domain sequence in the Pfam database. Predicted full-length sequences of key genes thought to be involved in the alliin synthetic pathway were then used for alignment.

### Analysis of alliin contents

Garlic root, bulb, inner bulb, sprout, and wounded leaf samples were prepared for analyses of alliin contents. Samples were ground in liquid nitrogen before adding 4% sulfosalicylic acid and ddH_2_O then stored at 25 °C for 30 min. Centrifugation at12,000 rap for 20 min was then performed and the supernatant was used for analysis of alliin content. To ensure the accuracy of the data, at least three replicates were obtained per tissue sample. Values represent the mean ± standard error.

### Statistical analysis

The Benjamini-Hochberg method was used to correct *p*-values. Adjusted p-values were then used to determine the False Discovery Rate (FDR), a key indicator of differentially expressed genes and an important method of reducing false-positive expression. All statistical analyses were carried out using SPSS software version 22.0. Differences were compared using one-way analysis of variance (ANOVA).

## Supplementary Information

**Additional file 1: Table S1**. Clean reads obtained from each sample group.

**Additional file 2: Figure S1**. Differential expression of KEGG pathway-related genes.

**Additional file 3: Figure S2**. Volcano plot of T1/T3 vs. T7/T9, T1/T3 vs. T7/9 and T1/T3 vs. T10/12 of upgenes and downgenes.

**Additional file 4: Figure S3**. The figure is unigenes contrasted to the COG database.

**Additional file 5: Table S2**. Cysteine-related GO terms.

**Additional file 6: Table S3**. Comparisons of cysteine pathway-related unigenes between T 1/3 vs. T 4/6, T 1/3 vs. T 7/9, and T 1/3 vs. T10/12.

**Additional file 7: Table S4**. Serine-related GO terms.

**Additional file 8: Table S5**. Comparisons of serine pathway-related unigenes between T 1/3 vs. T 4/6, T 1/3 vs. T 7/9, and T 1/3 vs. T10/12.

**Additional file 9: Table S6**. Sulfur-related GO terms.

**Additional file10: Table S7**. Comparisons of sulfur pathway-related unigenes between T 1/3 vs. T 4/6, T 1/3 vs. T 7/9, and T 1/3 vs. T10/12.

**Additional file 11: Table S8**. The eight candidate TFs involved in regulation of alliin synthesis in garlic.

## Data Availability

The datasets generated and analysed during the current study are available in the ENA Short Read Archive with accession number PRJEB33852.

## Abbreviations

Sykam S-433d: Amino acid measuring instrument
MCSO: S-methyl-L-cysteine sulfoxide
PCSO: S-propyl-L-cysteine sulfoxide
ACSO: S-allyl-L-cysteine sulfoxide
PECSO: S-trans-1-propenyl-L-cysteine sulfoxide
T1/3-T4/6: T01/02/03-T04/05/06
T1/3-T7/9: T01/02/03-T07/08/09
T1/3-T10/12: T01/02/03-T10/11/12
NR: Non-redundant protein
GO: Gene Ontology
COG: Clusters of Orthologous Groups of proteins
KOG: EuKaryotic Orthologous Groups
KEGG: Kyoto Encyclopedia of Genes and Genomes
CYS: Cysteine
SER: Serine
PW: Pizhou white garlic
