# Supplementary material for: Parallel analysis of global garlic gene expression and alliin content following leaf wounding
Source: BMC Plant Biol. 2021 Apr 10;21:174. doi: 10.1186/s12870-021-02948-0 (PMC8035738; doi:10.1186/s12870-021-02948-0)
Supplement: Supplementary file 2 — Additional file 2: Figure S1. Differential expression of KEGG pathway-related genes. [file 12870_2021_2948_MOESM2_ESM.doc]

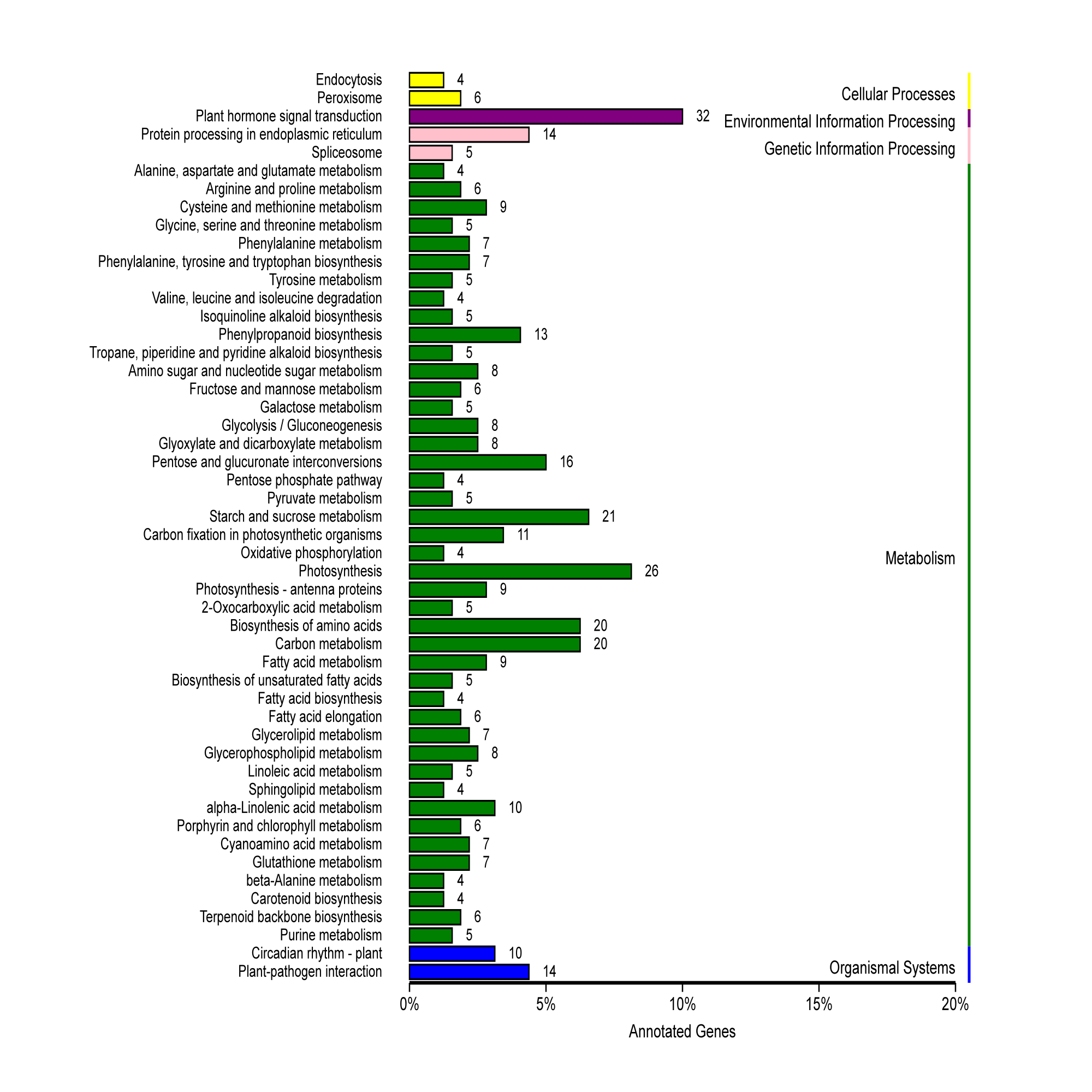

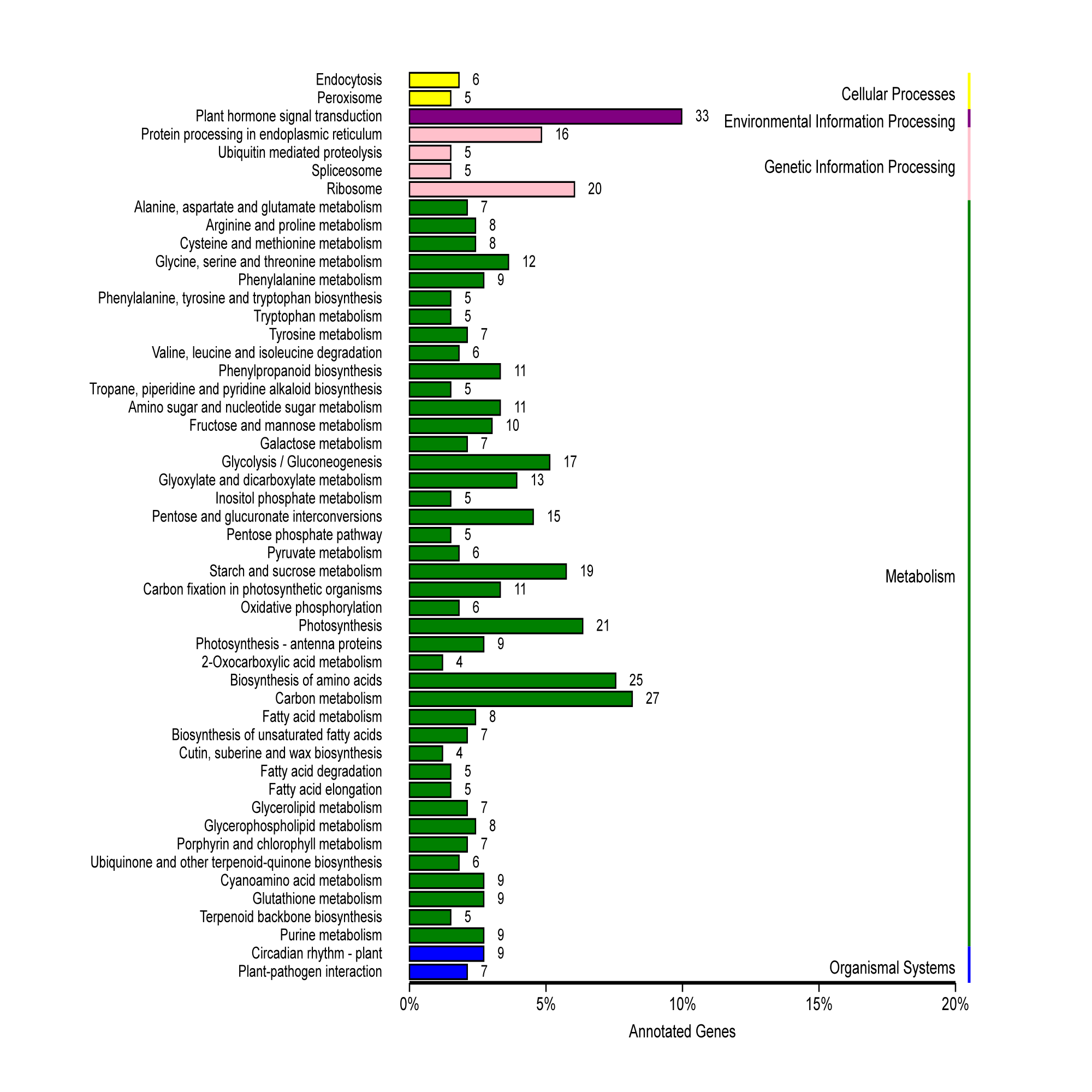


a

b

Figure S1 Classification of enriched KEGG terms. A large number of unigenes could be classified into various KEGG metabolic and signaling pathways. a T01_T02_T03_vs_T07_T08_T09 KEGG figure b T01_T02_T03_vs_T10_T11_T12 KEGG figure.
