# Supplementary material for: Parallel analysis of global garlic gene expression and alliin content following leaf wounding
Source: BMC Plant Biol. 2021 Apr 10;21:174. doi: 10.1186/s12870-021-02948-0 (PMC8035738; doi:10.1186/s12870-021-02948-0)
Supplement: Supplementary file 3 — Additional file 3: Figure S2. Volcano plot of T1/T3 vs. T7/T9, T1/T3 vs. T7/9 and T1/T3 vs. T10/12 of upgenes and downgenes. [file 12870_2021_2948_MOESM3_ESM.doc]

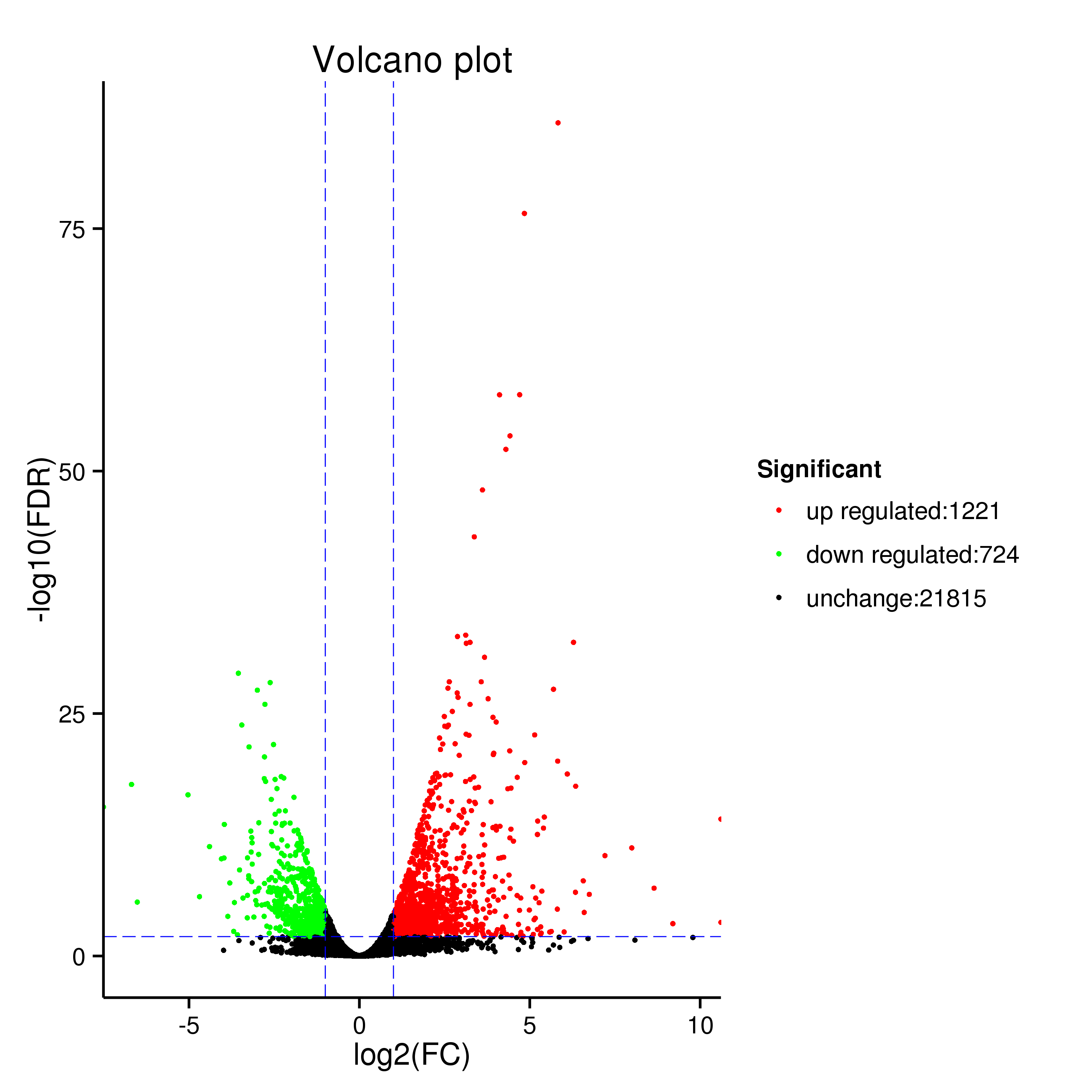


a


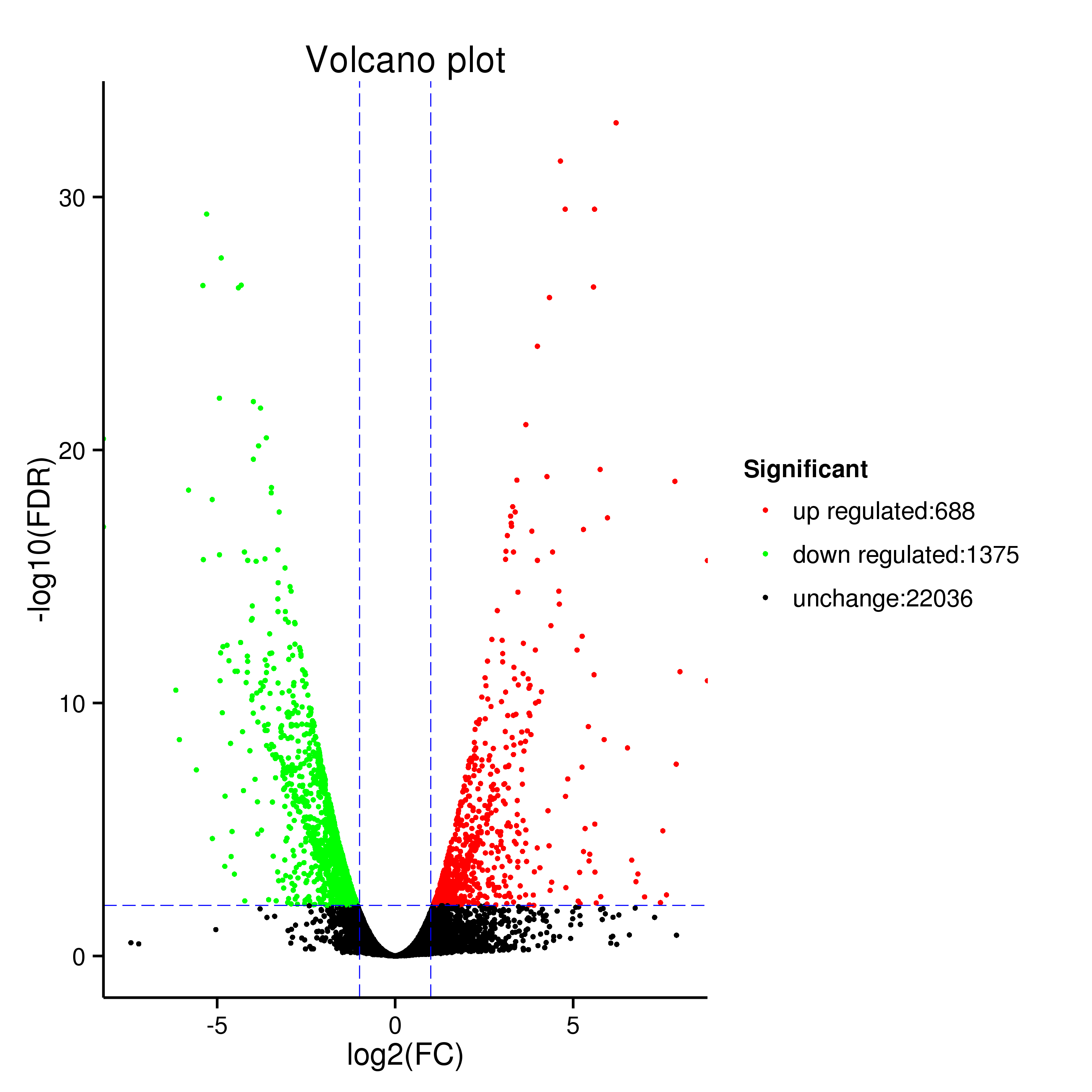


b

Figure S2 The difference of gene expression level between two groups of samples and its statistical significance. a is T01_T02_T03_vs_T07_T08_T09 Volcano Plot b isT01_T02_T03_vs_T10_T11_T12 Volcano Plot.
