# Supplementary material for: Parallel analysis of global garlic gene expression and alliin content following leaf wounding
Source: BMC Plant Biol. 2021 Apr 10;21:174. doi: 10.1186/s12870-021-02948-0 (PMC8035738; doi:10.1186/s12870-021-02948-0)
Supplement: Supplementary file 4 — Additional file 4: Figure S3. The figure is unigenes contrasted to the COG database. [file 12870_2021_2948_MOESM4_ESM.doc]

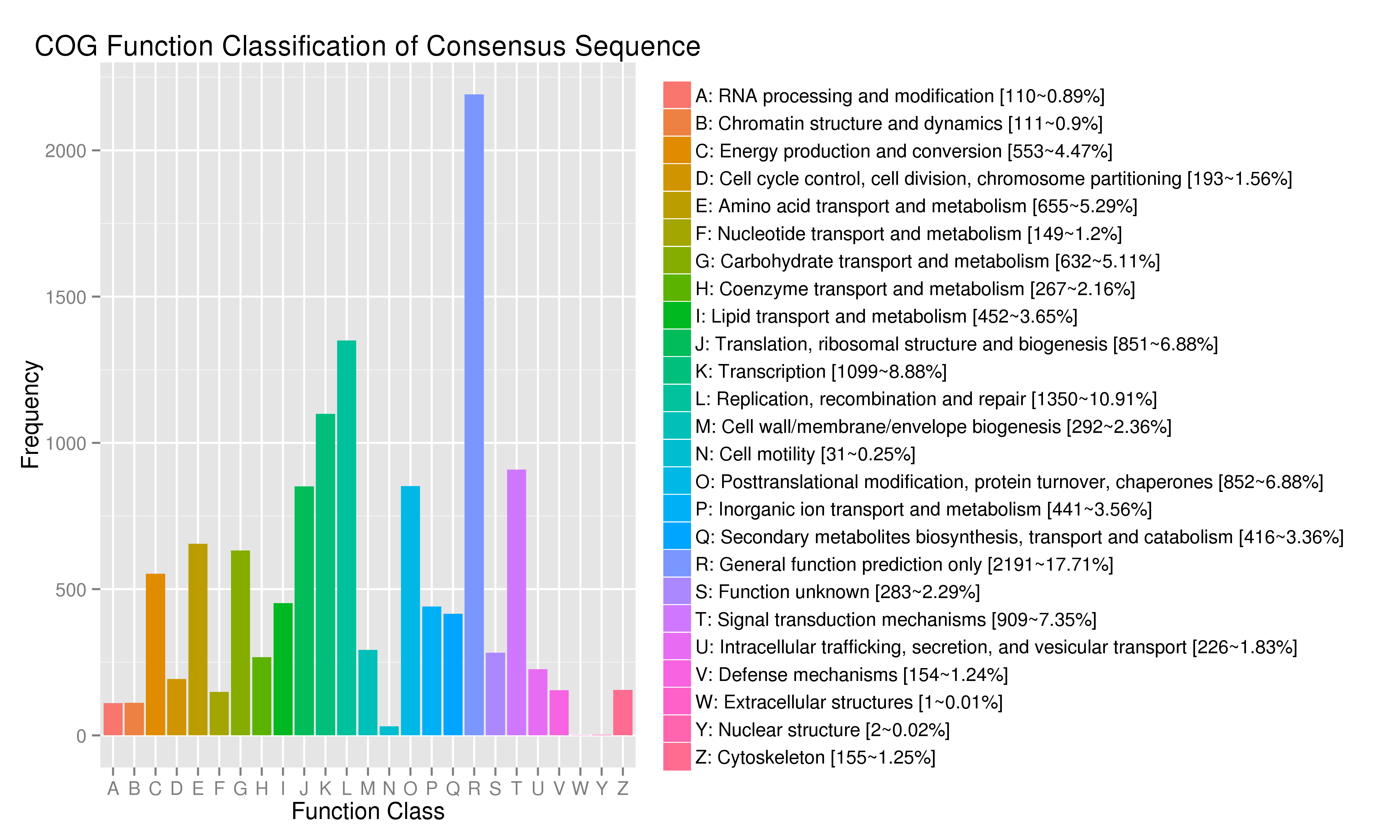


a

b


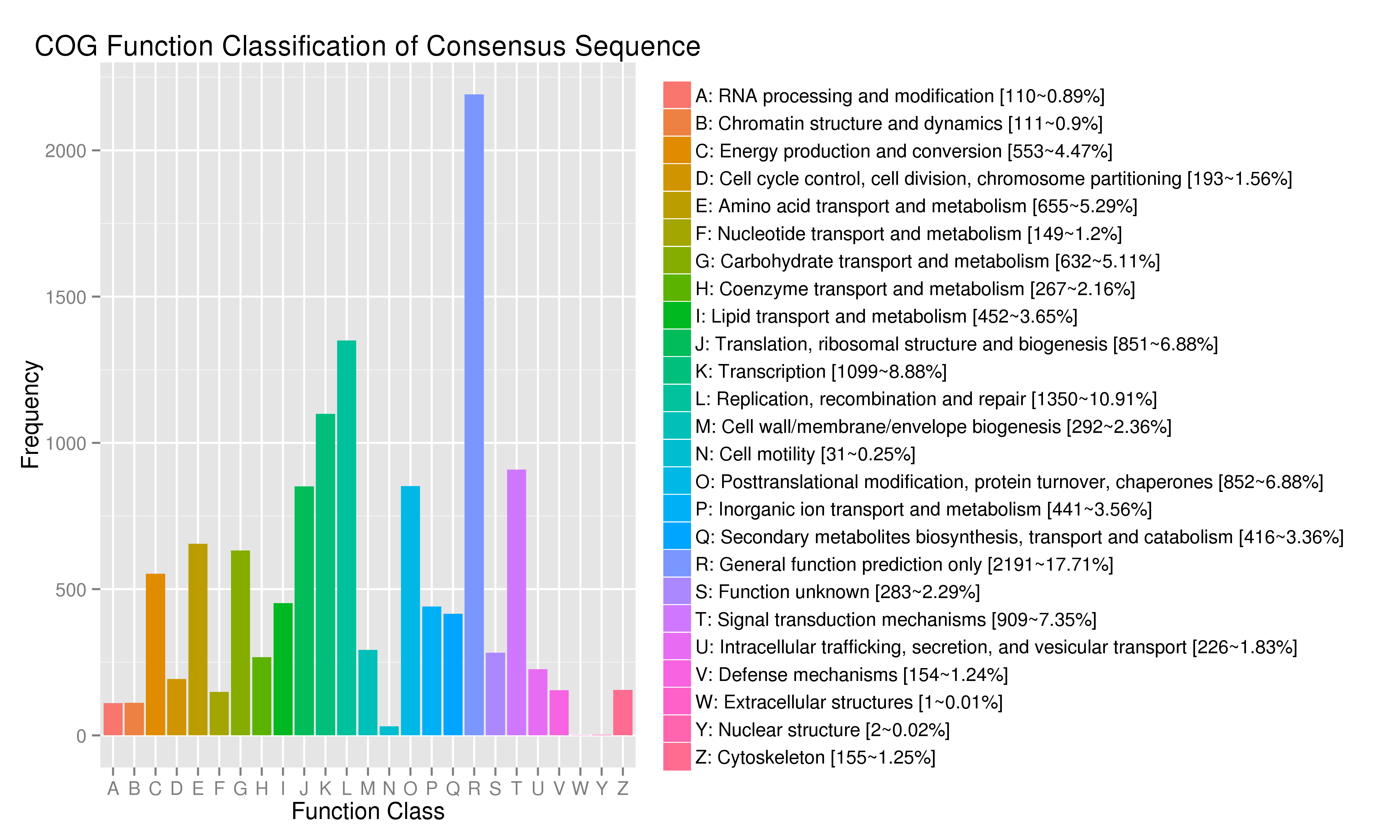


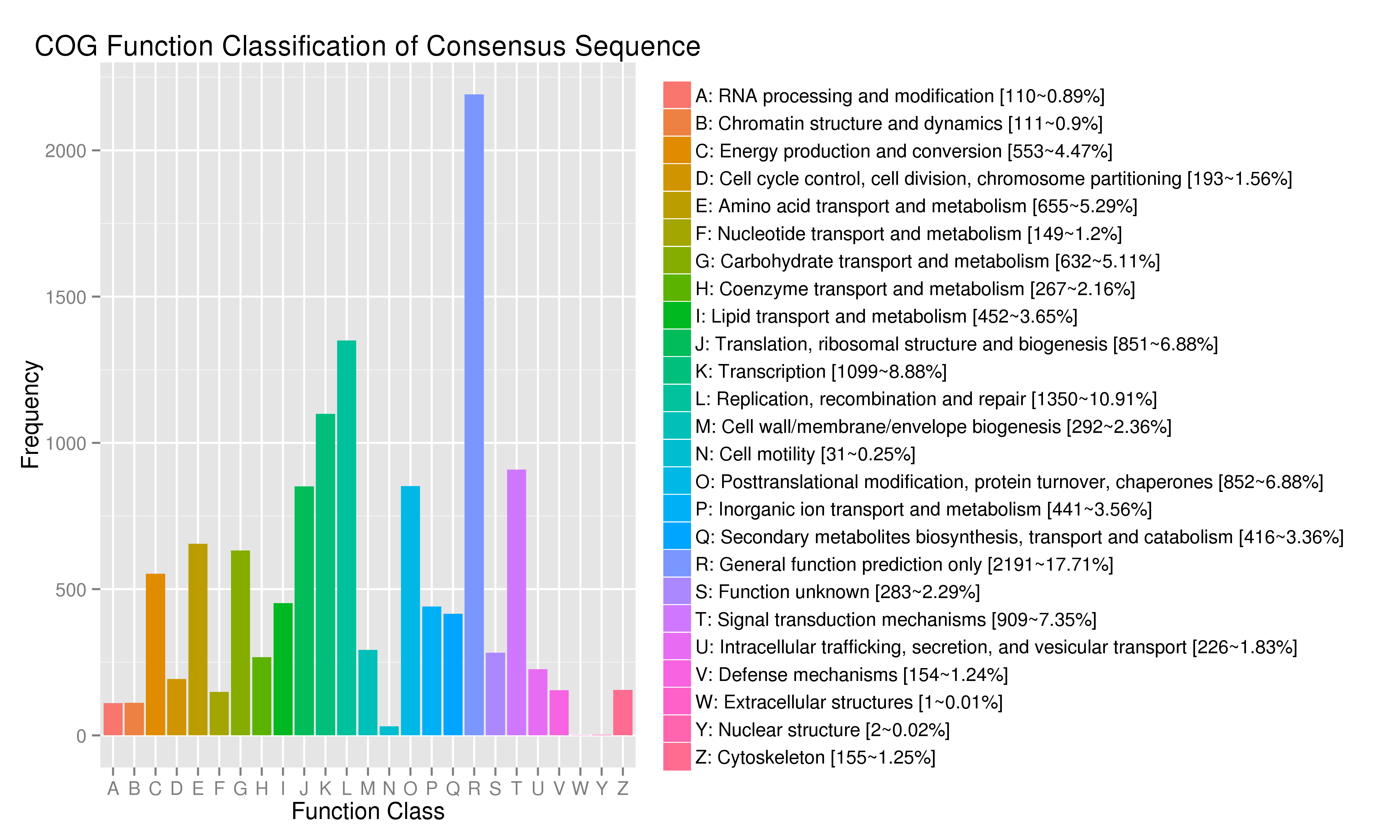


c

Figure S3 The figure is unigenes contrasted to the COG database. a T01_T02_T03_vs_T04_T05_T06 b T01_T02_T03_vs_T07_T08_T09

c T01_T02_T03_vs_T10_T11_T12
